# Characterization of Environmental Levels of Pesticide Residues in Household Air and Dust Samples near a Bioenergy Plant Using Treated Seed as Feedstock

**DOI:** 10.3390/ijerph20216967

**Published:** 2023-10-24

**Authors:** Jabeen Taiba, Eleanor G. Rogan, Daniel D. Snow, Chandran Achutan, Muhammad Zahid

**Affiliations:** 1 Department of Environmental, Agricultural and Occupational Health, College of Public Health, University of Nebraska Medical Center, Omaha, NE 68198-4388, USA; 2 Water Sciences Laboratory, University of Nebraska, Lincoln, NE 68583-0844, USA

**Keywords:** pesticides, neonicotinoids, indoor air, surface dust sampling

## Abstract

Exposure to neonicotinoid insecticides is associated with adverse human health outcomes. There is environmental contamination in Saunders County, Nebraska, due to the accumulation of fungicides and insecticides from a now-closed ethanol plant using seed corn as stock. A pilot study quantified environmental contamination in nearby houses from residual pesticides by measuring dust and air (indoor/outdoor) concentrations of neonicotinoids and fungicides at the study site (households within two miles of the plant) and control towns (20–30 miles away). Air (SASS^®^ 2300 Wetted-Wall Air Sampler) and surface dust (GHOST wipes with 4 × 4-inch template) samples were collected from eleven study households and six controls. Targeted analysis quantified 13 neonicotinoids, their transformation products and seven fungicides. Sample extracts were concentrated using solid phase extraction (SPE) cartridges, eluted with methanol and evaporated. Residues were re-dissolved in methanol–water (1:4) prior to analysis, with an Acquity H-Class ultraperformance liquid chromatograph (UPLC) and a Xevo triple quadrupole mass spectrometer. We compared differences across chemicals in air and surface dust samples at the study and control sites by dichotomizing concentrations above or below the detection limit, using Fisher’s exact test. A relatively higher detection frequency was observed for clothianidin and thiamethoxam at the study site for the surface dust samples, similarly for thiamethoxam in the air samples. Our results suggest airborne contamination (neonicotinoids and fungicides) from the ethanol facility at houses near the pesticide contamination.

## 1. Introduction

Pesticides are ubiquitous in the environment, and their byproducts may produce significant ecological effects within agroecosystem food webs and negatively impact human health (e.g., potential honeybee colony collapse, reproductive and developmental disruption, carcinogenesis) [1,2,3,4]. In the United States (U.S.), approximately 900 pesticides are registered for use and sold as more than 20,000 products [5]. Pesticides are also used as seed coatings which typically contain a mixture of at least one systemic neonicotinoid insecticide and several fungicides with different modes of action [6]. Despite the widespread use of treated seeds in the U.S., no federal laws govern the disposal of excess amounts of treated seeds [7].

**Figure 1:**
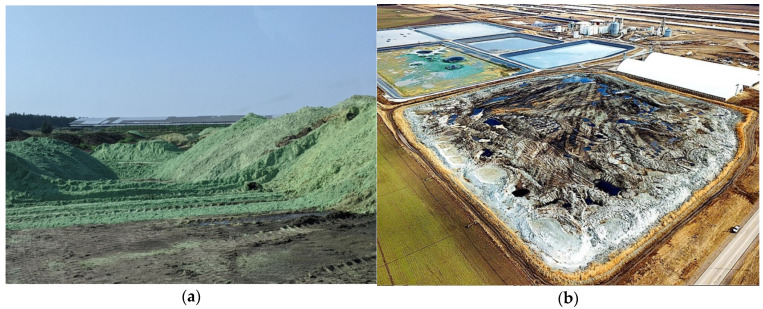
Piles of wet cake at the AltEn plant facility. (**a**) The green color comes from dye on the seeds. Courtesy: Judy Wu-Smart, 2021 [8]. (**b**) Drone view of AltEn plant product storage. In 2022, the piles of wet cake were moved to one 16-acre area off the site and covered with a concrete–clay shell. The brown color is liquid seepage from the piles. Courtesy: John Schalles, 2022 [9].

The AltEn ethanol plant near Mead, Nebraska, changed from purchased corn to discarded seed corn as its stock material in 2015. It produced 24 million gallons of ethanol yearly until it was closed by state officials in 2021 for non-compliance with state environmental regulations, as well as a four-million-gallon spill of sludge that spread through ditches and creeks [8]. During the seven years of using seed corn, pesticide-laden solid waste and wastewater accumulated at the plant site, resulting in the accumulation of 150,000,000 gallons of pesticide-laden wastewater stored in lagoons and over 84,000 tons of “wet cake” solid waste stored there (Figure 1a) [8]. In 2022, the piles of wet cake were moved to one 16-acre site and covered with cement and clay, as shown in Figure 1b [9]. As analyzed in 2019, the wet cake contained 42,700 parts per billion (ppb) of clothianidin and 81,500 ppb of thiamethoxam, about 85 times higher than the maximum amount permissible by law [9]. As early as 2017, residents around the plant started noticing a rotten smell and reported eye and throat irritation and nosebleeds. A researcher from the University of Nebraska-Lincoln detected a decline in bee health, which was attributed to neonicotinoid exposure [2]. In addition, the pesticides were detected in waterways south of the plant. 

Contaminated dust from seeds treated with neonicotinoids is bound to airborne soil particles, which can further contaminate surfaces and surface water [10]. Neonicotinoids are somewhat persistent, and clothianidin has the most prolonged half-life of soil degradation—545 days [11]. Several published studies have reported detection of neonicotinoids in indoor dust [12,13,14], finished drinking water [15] and human matrices, including blood and urine [16,17]. Previous studies have also demonstrated that some neonicotinoid metabolite toxicity is higher than their parent neonicotinoids in humans [18]. Additionally, in the general population, chronic environmental exposure to neonicotinoids has been associated with a variety of adverse human health effects [19,20,21,22,23,24,25]. Thus, it is essential to investigate the exposure levels for neonicotinoids and fungicides in residential indoor and outdoor environments near a pesticide-laden site.

**Figure 2:**
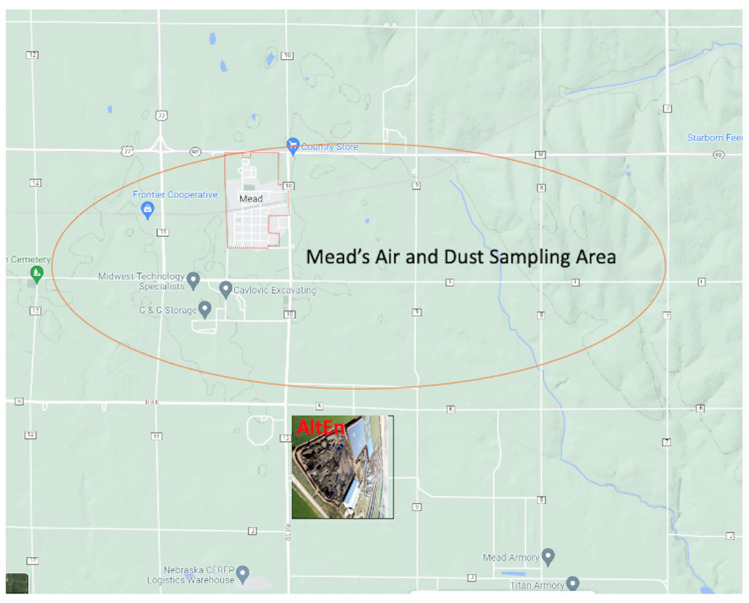
Map of the study site showing AltEn plant (rectangle) and the area (oval) where air and dust were sampled in 11 houses. Road numbers are given in small white boxes. Map was created using: https://www.google.com/maps/@41.2081164,-96.4626861,7447m/data=!3m1!1e3!5m1!1e4?entry=ttu (accessed on 2 August 2023).

This article presents the results from an environmental sampling pilot study conducted in households close to the contamination source (AltEn plant) and control sites. In this pilot study, we sampled the surface dust (*n* = 20) and air (*n* = 21) from eleven houses in the study site in Saunders County (Figure 2) and six control sites in neighboring counties (Washington and Douglas) to detect the characteristics and profiles of neonicotinoids and their metabolites, fungicides and an organophosphate insecticide in dust and air samples. These pesticide analytes included six parent neonicotinoids (acetamiprid, clothianidin, dinotefuran, imidacloprid, thiacloprid and thiamethoxam), seven neonicotinoid transformation products (6-chloronicotinic acid, 6-chloronicotinic aldehyde, 6-chloro-*N*-methylnicotinamide, imidacloprid desnitro, imidacloprid olefin, imidacloprid urea and thiamethoxam urea), fungicides (azoxystrobin, metalaxyl, picoxystrobin, pyraclostrobin and trifloxystrobin), a sulfoxamine insecticide (sulfoxaflor), an oxadiazine insecticide (indoxacarb) and one organophosphate insecticide (dimethoate). The neonicotinoids included in our analysis, imidacloprid, acetamiprid, clothianidin and thiacloprid, were previously biomonitored in the U.S. National Health and Nutrition Examination Survey (NHANES) 2015–2016 cycle [26].

## 2. Materials and Methods

### 2.1. Site Description

The AltEn ethanol plant is located near the town of Mead, Nebraska (population 617 in 2020). The study group was comprised of residences in Saunders County, located within a two-mile radius of the AltEn ethanol plant, as shown in Figure 2. The control group included residences in Kennard and Omaha, Nebraska, which are located 20 to 30 miles northeast of the ethanol plant. Kennard is an agricultural town of approximately 500 people, like Mead, whereas Omaha is distant from agricultural areas. 

### 2.2. Air and Dust Sampling

The air sampling was conducted using a SASS^®^ 2300 Wetted-Wall Air Sampler (Research International, Inc., Monroe, Washington, DC, USA). The air collection rate for this instrument is >300 L per minute (LPM), and it is suitable for a 0.5–10 µm particulate size range. The collection time for each sample was one hour, and each sample size was ~4–5 mL. 

Most of the houses we sampled are in the size range of 1300–1500 square foot (sqft). Assuming a 1500 sqft house, the total air volume occupied at one time is ~180,095 L. Our sampling method allowed us to collect samples via passing 325 LPM, with a total volume of 19,500 L of air. The sampling technique and time gave us a representation of approximately 11% of air for each sample. Since most residents spent most of their time in the living area, all our indoor air samples were collected in the living room. To avoid cross-contamination between samples, the air sampler reservoir water was changed with new filtered deionized water before every sample. Field blanks were prepared similarly for each site sampling. Surface dust swipe sampling was conducted using 15 cm × 15 cm GHOST wipes (CAT#4210, Environmental Express). The selected areas (inside the house and on the outside exterior wall) were wiped with a GHOST wipe using a 4 × 4-inch sampling template, a standardized method to sample dust and ensure the sampling was of a standardized area each time. We sampled areas that are not dusted routinely, for example, bookshelves in the living room. Each sample wipe was saved in a separate resealable tube, and to avoid cross-contamination, new latex gloves were used for each sampling. A field blank sample for each site was prepared similarly without touching any surface and saved in the same way. All samples were stored at −20 °C after collection. 

### 2.3. Sample Preparation and Analysis

Quantification of target pesticides in air and surface dust samples used ultraperformance liquid chromatography-tandem mass spectrometry (LCMS/MS) at the Water Science Laboratory (WSL), University of Nebraska, Lincoln, USA. Laboratory reagents and reference standards were purchased from Fisher Scientific (St. Louis, MO, USA), Sigma Aldrich (St. Louis, MO, USA) and Chemservice (West Chester, PA, USA)). Aqueous samples from the SASS device were weighed and spiked with three surrogates (10 ng each nitenpyram, terbuthylazine and dimoxystrobin), filtered through combusted glass microfiber filters (Whatman GF/A) and extracted using preconditioned 200 mg Oasis HLB (Waters Corporation, Milford, MA, USA) solid phase extraction cartridges, following similar procedures described elsewhere [27,28]. Cartridges were air-dried, eluted with 4 mL of methanol and 4 mL of acetonitrile (Optima Grade, Fisher Scientific, St. Louis, MO, USA), and spiked with 10 ng of labeled internal standards [(d3-clothianidin, d3-thiamethoxam, d4-imidacloprid, d6-metalaxyl, pyraclostrobin-(N-methoxy-d3), Cambridge Isotopes, Tewksbury, MA, USA]. GHOST wipes were weighed, quantitively divided, transferred to polypropylene centrifuge tubes, spiked with 25 ng of surrogates, and mixed with 5 mL of acetonitrile, following methods described elsewhere [29]. Tubes were shaken using a wrist-action shaker for 30 min and centrifuged at 3000 rpm for 10 min, and the solvent was transferred to an evaporation tube. Wipes were extracted a second time with 4 mL of acetonitile and 2.0 g of sodium chloride. Solvent portions were combined, filtered through 0.2 µm Teflon^TM^ syringe filters, evaporated using dry nitrogen at room temperature to near dryness and spiked with 10 ng labeled internal standards. Solvent extracts from both sample types were mixed with high purity ASTM Type I organic-free reagent water in the same proportion as the mobile phase and transferred to autosampler vials. Quality controls include synchronous analysis of laboratory reagent blanks, fortified blanks, laboratory duplicates and fortified matrix samples, each processed and analyzed at a rate of no less than 5% of all field samples. All extracts were analyzed by multiple reaction monitoring using a Xevo TQ-S Micro tandem quadrupole mass spectrometer interfaced with an Acquity H-Class UPLC separation system via a UniSpray^TM^ ionization source (Waters Corporation, Milford, MA, USA). Quantitation and confirmatory transitions were determined by infusing the reference standard. Instrument detection limits and method detection limits were determined using USEPA protocols [30].

### 2.4. Data Analysis

The targeted chemical values (µg/L) from the LCMS/MS system for air samples were normalized with the total volume of air passed through the system during sampling time by using the formula C=(C0/F) ’ T, where *C* is the analyte concentration in the air samples (µg/m^3^), *C*_0_ is the analyte concentration from the LCMS/MS (µg/L), F is the flowrate of the pump (L/min) and *T* is the sampling time (min). For the dust wipe samples, however, concentrations (ng/g) for all targeted chemicals are reported without any further conversion. 

All statistical analyses were conducted using SAS 9.4 software. The samples consisted of 20 dust and 21 air samples collected from 11 households from the study site and six control sites. Descriptive statistics include the mean, standard deviation, median and interquartile range (IQR) of all contaminants that had a value above the detection limit. All values were further dichotomized as to whether a sample had a concentration above the detection level or below the detection limit, using Fisher’s exact test to identify significant differences; *p*-values less than 0.05 were considered statistically significant.

## 3. Results

Both the air and dust samples were analyzed for twenty-one pesticides. Of these, seven target analytes, including acetamiprid, 6-chloronicotinicaldehyde, 6-chloro-N-methylnicotinamide, dimethoate, indoxacarb, sulfoxaflor and thiacloprid, were detected below the detection limit (0.004–0.009 µg/L) for air samples and (0.07–0.36 ng/g) for surface dust samples, respectively, and will not be discussed further. The mean, standard deviation, median and interquartile range for all of the analytes (azoxystrobin, clothianidin, 6-chloronicotinic acid, dinotefuran, imidacloprid, imidacloprid olefin, imidacloprid desnitro, imidacloprid urea, metalaxyl, picoxystrobin, pyraclostrobin, thiamethoxam and thiamethoxam urea) that were detected at or above the detection limit in the dust and air samples are summarized in Table 1, Table 2, Table 3 and Table 4. 

We observed a higher detection frequency for thiamethoxam in the air samples (indoor = 60%; outdoor = 60%) collected at the study site compared to other neonicotinoids (Table 5). We conducted Fisher’s exact test to measure the association between pesticides detected at or above the detection limit and the study or control groups for both air and surface dust samples. Clothianidin and thiamethoxam were detected more frequently at or above the detection limit for surface dust samples measured at the study site compared to the control sites (indoor and outdoor: *p*-value < 0.05). Among all of the analytes in the air samples, we found that thiamethoxam was detected at a significantly higher frequency at the study site compared to the control sites (indoor and outdoor: *p*-value < 0.05), as shown in Table 5. Additionally, the levels of pesticide detected were generally higher in the study site compared to the control sites for air and surface dust samples, as shown in Supplementary Material Figures S1 and S2. Among the neonicotinoids, a relatively higher detection frequency was observed for clothianidin (indoor = 100%; outdoor = 90%) and thiamethoxam (indoor = 100%; outdoor = 73%) in surface dust samples collected at the study site, and the mean and standard deviation values for clothianidin in the surface dust samples were higher for outdoor [14.15 μg/L (22.69)] than indoor [7.03 μg/L (5.63)] samples from the study site, as shown in Table 1, Table 2 and Table 5. 

Azoxystrobin was the fungicide detected most frequently in the surface dust samples (indoor = 73%; outdoor = 100%) collected at the study site. The mean and standard deviation values for azoxystrobin in the dust samples were also higher outdoors [11.83 μg/L (16.23)] than indoors [3.52 μg/L (4.49)] at the study site. 

Our results suggest that airborne contamination from the ethanol processing facility resulted in contamination of air and household dust in nearby houses. 

## 4. Discussion

The findings from this study show that parent and degradation product residues of clothianidin, dinotefuran, imidacloprid, imidacloprid olefin, imidacloprid desnitro, imidacloprid urea, thiamethoxam and thiamethoxam urea, and the fungicides, azoxystrobin, pyraclostrobin and metalaxyl, were detected in surface dust samples both inside and outside residences close to a pesticide-contaminated site, the ethanol plant. Clothianidin, imidacloprid, imidacloprid desnitro, thiamethoxam and 6-chloronicotinic acid and the fungicide azoxystrobin were detected in air samples above the limit of detection, particularly in houses in Mead. Moreover, in the dust samples, clothianidin and thiamethoxam showed a significantly higher detection frequency for the surface dust samples measured in houses at the study site compared to the control sites (indoor and outdoor: *p*-value < 0.05). 

Furthermore, a relatively higher detection frequency was observed for clothianidin (indoor = 100%; outdoor = 90%) and thiamethoxam (indoor = 100%; outdoor = 73%) in the surface dust samples collected at the study site. Our results are comparable to a few Chinese indoor dust sample studies, in which acetamiprid and imidacloprid were the primary target analytes detected in 98.8% and 99.7% of samples, respectively [3], and 98% of samples in a nationwide study [31]. The degradation product *N*-desmethyl acetamiprid has been detected in 56% of indoor dust samples [14], and other residues have also been detected in indoor dust samples [8,13,14,31,32]. Seed treatment pesticides and their metabolites have been associated with neurotoxicity and carcinogenicity in humans [1,33], changes in insulin and glucose metabolism, and hematological homeostasis [23,24].

Among all of the analytes in the air samples, we found a significantly higher detection frequency for thiamethoxam in houses at the study site compared to the control sites (indoor and outdoor: *p*-value < 0.05). Azoxystrobin had a higher detection frequency in the surface dust samples (indoor = 73%; outdoor = 100%) collected at study site houses. Our results are comparable to an indoor dust study in China, where strobilurin fungicides, including azoxystrobin, were detected in indoor dust samples (65.8–97.7%) [34]. Recent studies have shown that fungicides may pose adverse effects for animals and humans, including neurotoxicity [35], endocrine disrupting effects [36] and carcinogenicity [37].

Only clothianidin and thiamethoxam were detected significantly more frequently above the detection limit in the study group houses compared to the control group houses in the surface dust samples, and thiamethoxam was detected more in the air samples measured at the study site compared to the control sites (indoor and outdoor: *p*-value < 0.05). Our findings are important because clothianidin and thiamethoxam are commonly used in seed treatment. We suggest that these potentially significantly greater differences between the study and control groups among the surface dust samples are due to airborne contaminants in pesticide dust from the site of the AltEn ethanol plant. 

Other reasons for the higher detection of these neonicotinoids can be due to their persistence in water and soils (aqueous dissipation half-lives of 4.7–40.3 days; soil degradation half-lives of 3 to >1000 days), and non-volatility (<0.002 mPa at 25 °C), allowing them to be transported away from the area of application to different environmental areas [38]. Clothianidin and thiamethoxam have a soil persistence of 13–1386 days, which is 7–72 days higher than other neonicotinoids, such as acetamiprid (2–20 days) and thiacloprid (9–27 days) [39]. Though the concentration of clothianidin appears to decrease in agricultural soils after 4–6 years of treated seed use [40], these neonicotinoids can be detected several years after ceasing to use the treated seed [41]. 

Pesticides have been shown to migrate into houses during or after their application, through human or animal outdoor-indoor activities [42]. Pesticides are known to travel in the atmosphere, with long-range atmospheric transport and deposition from their emission area [43]. It is essential to assess the contamination of pesticides in the indoor environment, as there is a potential for human exposure via ingestion and inhalation of indoor dust [44].

This study is the first to investigate the airborne transport of pesticides from a pesticide-contaminated ethanol plant site into nearby residences. Because the plant is now closed, it is clear that transport on people, clothing or equipment is not occurring. The higher levels of pesticides and the higher frequencies of their detection in houses near the plant, compared to levels and frequencies found in houses in a similar small agricultural town (Kennard), indicate that the contamination of houses in Mead is from the pesticides stored on the plant site. 

Limited studies, primarily in China, have assessed the occurrence of pesticide residues (neonicotinoids) in indoor house dust [13,14,32,45]. One strength of this study is the documentation of the occurrence of pesticide residues in dust and air samples in residential settings. The limitations, however, include assessment of the indoor and outdoor pesticide contaminants in air and dust surfaces but not in human matrices (urine and blood). Very limited studies have reported any associations of neonicotinoids with human health. According to the US EPA (Environmental Protection Agency) toxicology report on clothianidin, the aggregate risk assessments based on acute and chronic dietary exposure from surface water are 3.97 and 2.14 ppb for the general U.S. population [46].

(1) $$\sum IMI\;RPF,i=\sum \left(NEOi\times RPFi\right)=imidacloprid+acetamiprid\times 0.8+thiamethoxam\times 9.5+clothianidin\times 5.8.$$

And based on the recommended exposure assessment models in the US Exposure Factors Handbook, the total average daily dose via inhalation was estimated using the following Equation (2):

(2) $$\sum_{\;}^{\;}ADDinh,\;total\;=\sum_{\;}^{\;}ADDinh,i\;=\;\sum_{\;}^{\;}\left(IMI\;RPF,i\;\;\times \;IR\;\times \;EF\;\times \;\frac{ED}{BW\;}\;\times AT\right)$$

where *IMI_RPF,i_* is the contaminant concentration in inhaled air from Equation (1); *IR* is the inhalation rate (m^3^ day^−1^); *EF* is the exposure frequency (days); *ED* is the exposure duration (years); *BW* is the body weight (kg); and *AT* is the average time [47]. 

A second limitation is that our sample size was limited in this pilot study; thus, the measured concentration of each target analyte in air and surface dust samples could be over-or under-estimated.

## 5. Conclusions

In conclusion, neonicotinoids and fungicides detected in residences near a bioenergy plant that are at or above the detection limit are attributable to environmental contaminants stored at the plant. Furthermore, as exposure to residues poses various adverse human health effects, additional exposure studies in residential settings measuring indoor house dust will be important, as it is a significant pathway for human exposure.

## Figures and Tables

**Table 1:** Descriptive statistics for neonicotinoid analytes detected at or above the detection limit in indoor and outdoor surface dust samples.

		Indoor Dust		Outdoor Dust	
Contaminant		**Study**	**Control**	**Study**	**Control**
Clothianidin		(N = 11)	(N = 3)	(N = 10)	(N = 1)
	Mean (SD)	7.03 (5.63)	1.42 (1.14)	14.15 (22.69)	0.17
	Median (IQR)	5.06 (2.40, 11.58)	1.81 (0.14, 2.31)	3.23 (0.24, 14.54)	0.17 (0.17, 0.17)
Dinotefuran		(N = 6)	(N = 2)	(N = 2)	(N = 2)
	Mean (SD)	1.90 (2.83)	2.26	0.15	0.42
	Median (IQR)	0.67 (0.25, 2.06)	2.26 (0.32, 4.21)	0.15 (0.15, 0.15)	0.42 (0.17, 0.68)
Imidacloprid		(N = 5)	(N = 4)	(N = 3)	(N = 2)
	Mean(SD)	1.57 (1.63)	0.38 (0.15)	1.39 (1.90)	3.09
	Median (IQR)	1.48 (0.23, 1.77)	0.38 (0.26, 0.49)	0.45 (0.15, 3.58)	3.09 (0.12, 6.05)
Imidacloprid Olefin *		(N = 0)	(N = 0)	(N = 0)	(N = 1)
	Mean (SD)				0.44
	Median (IQR)				0.44 (0.44, 0.44)
Imidacloprid Desnitro *		(N = 4)	(N = 0)	(N = 1)	(N = 1)
	Mean (SD)	0.33 (0.12)		0.37	0.32
	Median (IQR)	0.28 (0.28, 0.40)		0.37 (0.37, 0.37)	0.32 (0.32, 0.32)
Imidacloprid Urea *		(N = 2)	(N = 0)	(N = 1)	(N = 1)
	Mean (SD)	0.39 (0.14)		0.38 (0)	0.32 (0)
	Median (IQR)	0.39 (0.29, 0.50)		0.38 (0.38, 0.38)	0.32 (0.32, 0.32)
Thiamethoxam		(N = 11)	(N = 2)	(N = 8)	(N = 1)
	Mean (SD)	1.76 (1.71)	1.13 (1.00)	3.19 (4.47)	0.09 (0)
	Median (IQR)	1.23 (0.52, 2.88)	1.13 (0.43, 1.84)	0.59 (0.15, 5.98)	0.09 (0.09, 0.09)
Thiamethoxam Urea *		(N = 0)	(N = 0)	(N = 3)	(N = 0)
	Mean (SD)			0.65 (0.44)	
	Median (IQR)			0.55 (0.26, 1.13)	

* Indicates unique data which are not reported as mean and do not have a standard deviation.

**Table 2:** Descriptive statistics for fungicide analytes detected at or above the detection limit in indoor and outdoor surface dust samples.

		Indoor Dust		Outdoor Dust	
Contaminant		**Study**	**Control**	**Study**	**Control**
Azoxystrobin		(N = 8)	(N = 1)	(N = 11)	(N = 5)
	Mean (SD)	3.52 (4.49)	0.53	11.83 (16.23)	4.09 (3.02)
	Median (IQR)	1.15 (0.67, 5.74)	0.53 (0.53, 0.53)	7.28 (3.05, 13.60)	4.78 (1.71, 5.68)
Metalaxyl *		(N = 1)	(N = 0)	(N = 2)	(N = 0)
	Mean (SD)	1.74 (.)		0.33 (0.08)	
	Median (IQR)	1.74 (1.74, 1.74)		0.33 (0.27, 0.39)	
Pyraclostrobin *		(N = 2)	(N = 0)	(N = 2)	(N = 2)
	Mean (SD)	2.55 (2.96)		0.61 (0.31)	1.70 (0.53)
	Median (IQR)	2.55 (0.45, 4.64)		0.61 (0.39, 0.83)	1.70 (1.33, 2.08)

* Indicates unique data which are not reported as mean and do not have a standard deviation.

**Table 3:** Descriptive statistics for neonicotinoid analytes detected at or above the detection limit in indoor and outdoor air samples.

		Indoor Air		Outdoor Air	
Contaminant		**Study**	**Control**	**Study**	**Control**
6-Chloronicotinic acid *		(N = 0)	(N = 2)	(N = 0)	(N = 1)
	Mean (SD)		0.09 (0.02)		0.02 (0)
	Median (IQR)		0.09 (0.07, 0.11)		0.02 (0.02, 0.02)
Clothianidin		(N = 10)	(N = 4)	(N = 9)	(N = 5)
	Mean (SD)	0.19 (0.27)	0.02 (0.01)	0.12 (0.17)	0.02 (0.01)
	Median (IQR)	0.05 (0.04, 0.21)	0.02 (0.01, 0.03)	0.05 (0.04, 0.06)	0.02 (0.01, 0.02)
Imidacloprid *		(N = 2)	(N = 1)	(N = 2)	(N = 0)
	Mean (SD)	0.07 (0.03)	0.04 (0)	0.05 (0.01)	
	Median (IQR)	0.07 (0.05, 0.09)	0.04 (0.04, 0.04)	0.05 (0.04, 0.06)	
Imidacloprid Desnitro *		(N = 1)	(N = 0)	(N = 2)	(N = 0)
	Mean (SD)	0.02 (0)		0.02 (0.01)	
	Median (IQR)	0.02 (0.02, 0.02)		0.02 (0.01, 0.02)	
Thiamethoxam *		(N = 6)	(N = 0)	(N = 6)	(N = 0)
	Mean (SD)	0.09 (0.03)		0.11 (0.10)	
	Median (IQR)	0.09 (0.07, 0.10)		0.06 (0.04, 0.19)	

* Indicates unique data which are not reported as mean and do not have a standard deviation.

**Table 4:** Descriptive statistics for fungicide analytes detected at or above the detection limit in indoor and outdoor air samples.

		Indoor Air		Outdoor Air	
Contaminant		**Study**	**Control**	**Study**	**Control**
Azoxystrobin		(N = 5)	(N = 2)	(N = 6)	(N = 1)
	Mean (SD)	0.15 (0.25)	0.02 (0)	0.19 (0.20)	0.02 (0)
	Median (IQR)	0.03 (0.02, 0.09)	0.02 (0.01, 0.02)	0.13 (0.06, 0.20)	0.02 (0.02, 0.02)
Picoxystrobin *		(N = 1)	(N = 0)	(N = 0)	(N = 0)
	Mean (SD)	0.01 (0)			
	Median (IQR)	0.01 (0.01, 0.01)			

* Indicates unique data which are not reported as mean and do not have a standard deviation.

**Table 5:** Study vs. Control (a) Dust and (b) Air samples: Comparing the percentage of samples at/above vs. below the detection limit.

			Indoor Dust		Outdoor Dust		
Contaminant		**Study**	**Control**	***p*** **-value**	**Study**	**Control**	***p*** **-value**
Clothianidin		(N = 11)	(N = 6)		(N = 11)	(N = 6)	
	≥DL, n (%)			**0.0294 ^1^**			**0.0054 ^1^**
	No	0 (0.0%)	3 (50%)		1 (9.1%)	5 (83.3%)	
	Yes	11 (100%)	3 (50%)		10 (90.9%)	1 (16.7%)	
Thiamethoxam		(N = 11)	(N = 6)		(N = 11)	(N = 6)	
	≥DL, n (%)			**0.0063 ^1^**			**0.0498 ^1^**
	No	0 (0.0%)	4 (66.7%)		3 (27.3%)	5 (83.3%)	
	Yes	11 (100%)	2 (33.3%)		8 (72.7%)	1 (16.7%)	
Azoxystrobin		(N = 11)	(N = 6)		(N = 11)	(N = 6)	
	≥DL, n (%)			**0.0498 ^1^**			0.3529 ^1^
	No	3 (27.3%)	5 (83.3%)		0 (0.0%)	1 (16.7%)	
	Yes	8 (72.7%)	1 (16.7%)		11 (100%)	5 (83.3%)	
Contaminant			**Indoor Air**		**Outdoor Air**		
Thiamethoxam		(N = 10)	(N = 6)		(N = 10)	(N = 6)	
	≥DL, n (%)			**0.0338 ^1^**			**0.0338 ^1^**
	No	4 (40.0%)	6 (100%)		4 (40.0%)	6 (100%)	
	Yes	6 (60.0%)	0 (0.0%)		6 (60.0%)	0 (0.0%)	

^1^ Fisher’s exact test *p*-value; Detection Limit (DL).

## Data Availability

The data that support the findings of this study are available on request from the corresponding author, M.Z.

## Supplementary Materials

The following supporting information can be downloaded at: https://www.mdpi.com/article/10.3390/ijerph20216967/s1, Figure S1: Detection of neonicotinoids and fungicides in Study and Control Groups’ Residential Indoor and Outdoor Surface Dust Samples; Figure S2: Detection of neonicotinoids and fungicides in Study and Control Groups’ Residential Indoor and Outdoor Air Samples.

## Abbreviations

EPA—Environmental Protection Agency, IQR—Interquartile Range, LPM—Liters per Minute, NHANES—National Health and Nutrition Examination Survey, RPF—Relative Potency Factor, UPLC—Ultraperformance Liquid Chromatography, WSL—Water Science Laboratory.
