# Supplementary material for: Characterization of Environmental Levels of Pesticide Residues in Household Air and Dust Samples near a Bioenergy Plant Using Treated Seed as Feedstock
Source: Int J Environ Res Public Health. 2023 Oct 24;20(21):6967. doi: 10.3390/ijerph20216967 (PMC10648468; doi:10.3390/ijerph20216967)
Supplement: Supplementary file 1 [file ijerph-20-06967-s001.zip › ijerph-2566801-supplementary.pdf]

Supplementary Materials:

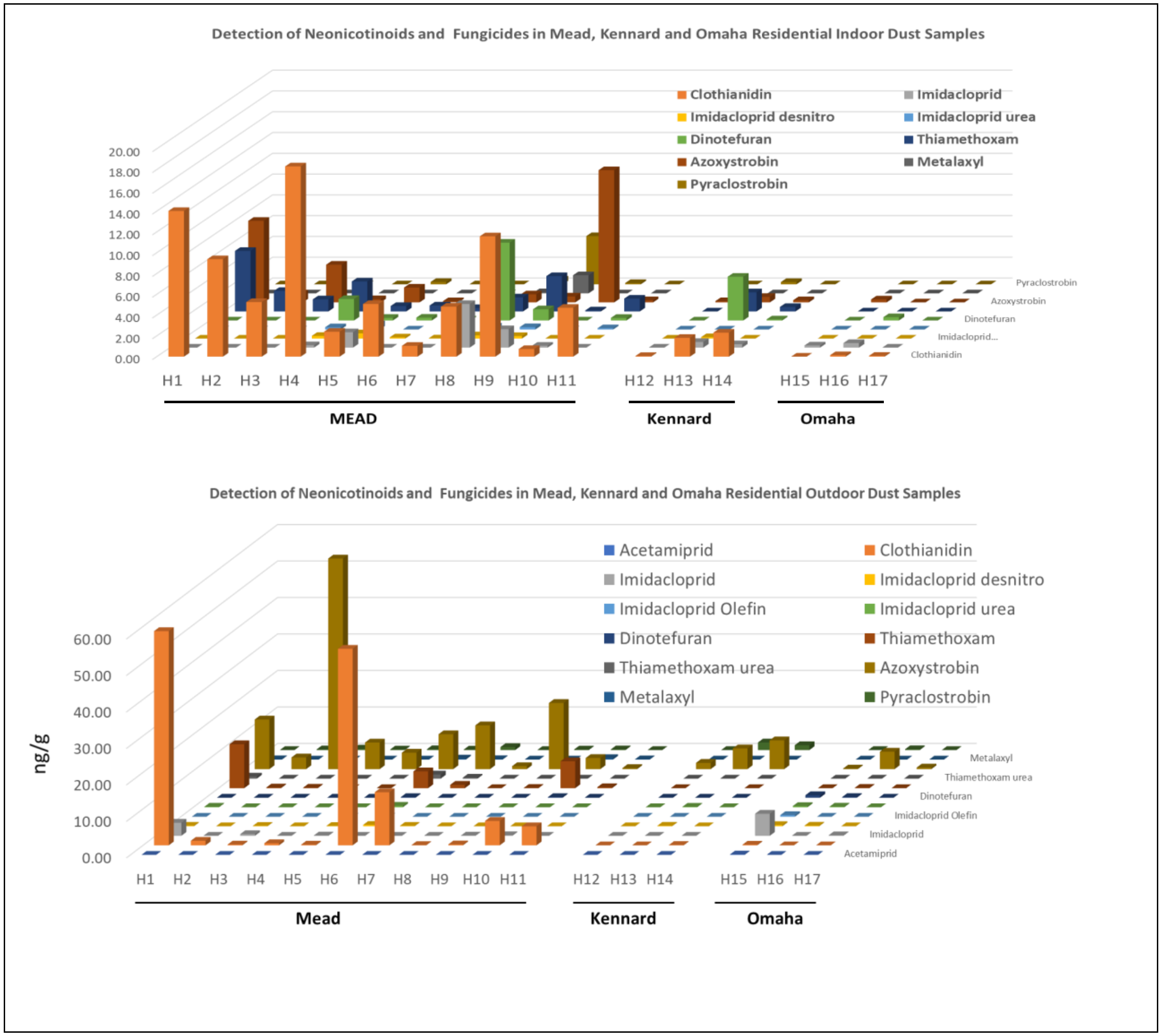

Figure S1. Detection of neonicotinoids and fungicides in Study and Control Group Residential Indoor and Outdoor Surface Dust Samples.

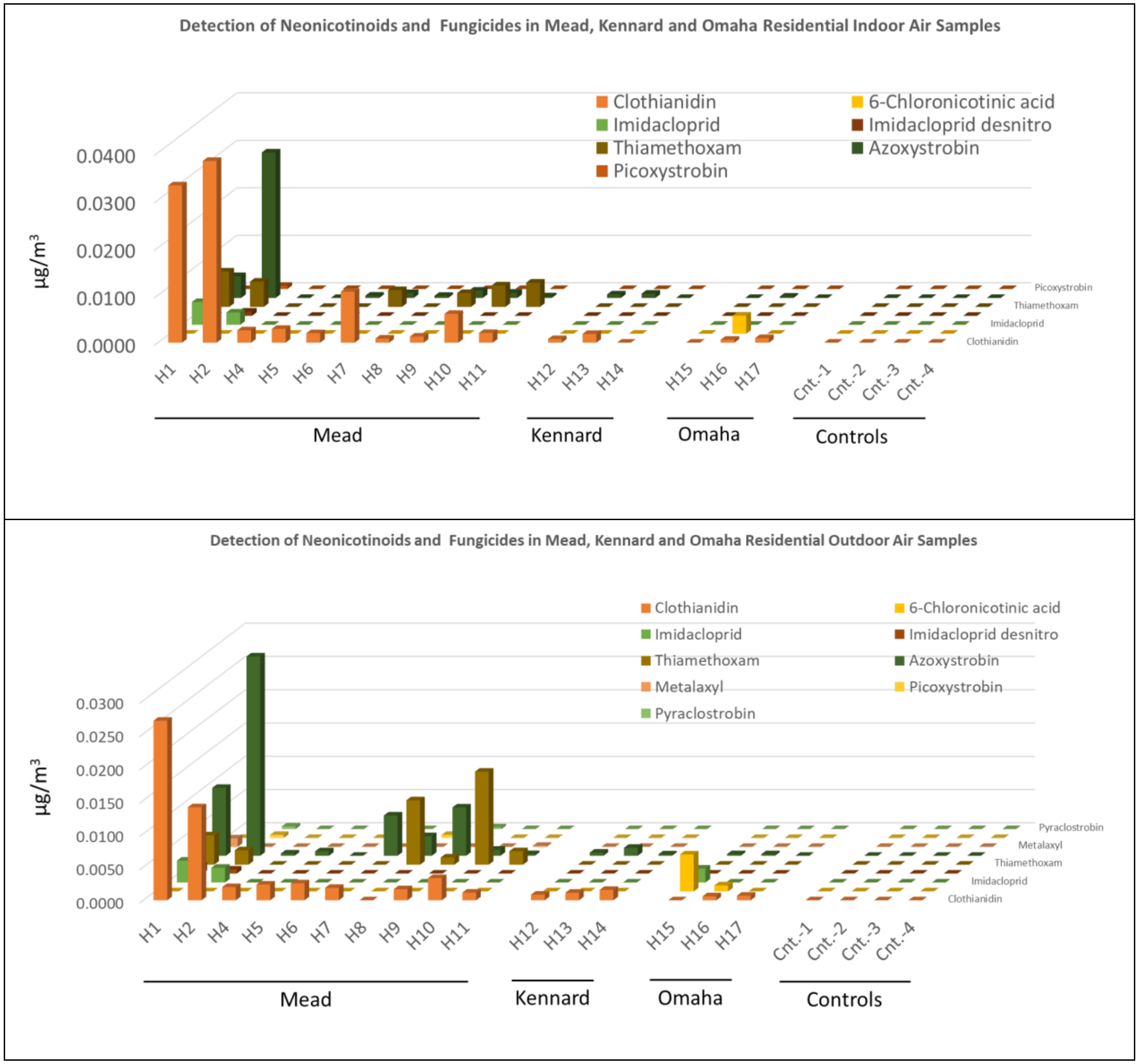

Figure S2. Detection of neonicotinoids and fungicides in Study and Control Groups' Residential Indoor and Outdoor Air Samples.

Raw data: Indoor and Outdoor dust sample data related to figure S1

Code id	Sample_ ID	Collection_ Date	Acetamiprid	Clothianidin	6-chloronicotinic aldehyde	6-chloro-N-methylnicotinamide	Imidacloprid	Imidacloprid desnitro	Imidacloprid Olefin	Imidacloprid urea	Indoxacarb	Dinotefuran	Thiacloprid	Thiamethoxam	Thiamethoxam urea	Dimethoate	Azoxystrobin	Trifloxystrobin	Metalaxyl	Picoxystrobin	Pyraclostrobin	Sulfoxaflor	%Dimoxystrobin	%Terbutylazine
H1-1	MH1-I	7/7/22	0.08	14.02	0.00	0.00	0.00	0.00	0.00	0.00	0.00	0.00	0.00	5.83	0.00	0.00	7.85	0.00	0.04	0.00	0.03	0.00	74.2	59.5
H1-2	MH1-O	7/7/22	0.00	58.72	0.00	0.00	3.58	0.14	0.00	0.13	0.00	0.00	0.00	12.04	0.55	0.00	13.60	0.00	0.08	0.00	0.00	0.00	94.7	79.8
H2-I	MH2-I	7/7/22	0.00	9.38	0.00	0.00	0.00	0.01	0.00	0.00	0.00	0.00	0.00	1.97	0.00	0.00	0.87	0.00	0.00	0.00	0.00	0.00	98.3	98.5
H2-2	MH2-O	7/7/22	0.00	1.25	0.00	0.00	0.00	0.01	0.00	0.00	0.00	0.00	0.00	0.18	0.00	0.00	3.20	0.09	0.00	0.01	0.18	0.00	104.9	98.1
H3-I	MH3-I	7/7/22	0.00	5.25	0.00	0.00	0.00	0.00	0.00	0.00	0.00	0.00	0.00	1.17	0.00	0.00	3.63	0.00	0.00	0.00	0.03	0.00	99.0	65.5
H3-2	MH3-O	7/7/22	0.00	0.22	0.00	0.00	0.45	0.10	0.00	0.09	0.00	0.00	0.00	0.12	0.00	0.00	57.72	0.05	0.00	0.00	0.39	0.00	104.5	98.3
H4-1	MH4-I	9/1/22	0.00	18.31	0.00	0.00	0.23	0.26	0.00	0.21	0.00	2.06	0.00	2.88	0.00	0.00	0.31	0.00	0.00	0.00	0.00	0.00	33.9	35.3
H4-2	MH4-O	9/1/22	0.00	0.58	0.00	0.00	0.00	0.07	0.00	0.06	0.00	0.00	0.00	0.05	0.00	0.00	7.28	0.03	0.00	0.00	0.15	0.00	96.6	91.8

H5-1	MH5-I	9/7/22	0.00	2.40	0.00	0.00	1.48	0.50	0.00	0.50	0.00	0.25	0.00	0.52	0.00	0.00	1.43	0.04	0.05	0.00	0.23	0.00	99.5	96.7
H5-2	MH5-O	9/7/22	0.00	0.18	0.00	0.00	0.07	0.08	0.00	0.06	0.00	0.00	0.00	0.00	0.00	0.00	4.50	0.05	0.00	0.00	0.23	0.00	114.4	105.9
H6-1	MH6-I	9/7/22	0.00	5.06	0.00	0.00	0.00	0.11	0.00	0.09	0.00	0.27	0.00	0.57	0.00	0.00	0.12	0.00	0.00	0.00	0.00	0.00	60.5	60.1
H6-2	MH6-O	9/7/22	0.00	53.87	0.00	0.00	0.00	0.37	0.00	0.38	0.00	0.15	0.00	4.61	1.13	0.00	9.56	0.00	0.27	0.00	0.12	0.00	54.3	77.1
H7-1	MH7-I	9/9/22	0.00	1.04	0.00	0.00	0.00	0.02	0.00	0.00	0.00	0.09	0.00	0.33	0.00	0.00	0.18	0.00	0.00	0.00	0.02	0.00	49.5	43.9
H7-2	MH7-O	9/9/22	0.00	14.54	0.00	0.00	0.00	0.11	0.00	0.10	0.00	0.00	0.00	0.92	0.26	0.00	12.00	0.00	0.09	0.00	0.83	0.00	62.8	54.9
H8-1	MH8-I	9/9/22	0.00	4.81	0.00	0.00	4.19	0.31	0.00	0.29	0.00	7.49	0.00	1.35	0.00	0.00	0.77	0.00	0.11	0.00	0.45	0.00	44.1	63.0
H8-2	MH8-O	9/9/22	0.00	0.06	0.00	0.00	0.04	0.02	0.00	0.02	0.00	0.00	0.00	0.00	0.00	0.00	0.81	0.05	0.00	0.00	0.05	0.00	11.2	10.5
H9-1	MH9-I	9/9/22	0.00	11.58	0.00	0.00	1.77	0.24	0.00	0.23	0.00	1.08	0.00	3.40	0.00	0.00	0.57	0.00	1.74	0.00	4.64	0.00	65.8	52.7
H9-2	MH9-O	9/9/22	0.00	0.24	0.00	0.00	0.05	0.06	0.00	0.05	0.00	0.00	0.00	0.07	0.00	0.00	18.13	0.05	0.00	0.06	0.14	0.00	79.2	75.1
H10-1	MH10-I	9/22/22	0.00	0.73	0.00	0.00	0.18	0.09	0.00	0.08	0.00	0.05	0.00	0.10	0.00	0.00	12.72	0.02	0.00	0.00	0.12	0.00	95.7	93.6
H10-2	MH10-O	9/22/22	0.00	6.69	0.00	0.00	0.15	0.02	0.00	0.02	0.00	0.15	0.00	7.36	0.05	0.00	3.05	0.00	0.39	0.00	0.18	0.00	44.7	28.8
H11-1	MH11-I	9/22/22	0.00	4.70	0.00	0.00	0.00	0.09	0.00	0.12	0.00	0.25	0.00	1.23	0.00	0.00	0.21	0.00	0.00	0.00	0.00	0.00	18.3	47.5
H11-2	MH11-O	9/22/22	0.00	5.22	0.00	0.00	0.00	0.02	0.00	0.00	0.00	0.00	0.00	0.26	0.00	0.00	0.29	0.00	0.07	0.00	0.00	0.00	44.1	57.1
H12-1	KH1-I	9/23/22	0.00	0.06	0.00	0.00	0.00	0.00	0.00	0.00	0.00	0.00	0.00	0.00	0.00	0.00	0.12	0.00	0.00	0.00	0.00	0.00	54.6	61.3
H12-2	KH1-O	9/23/22	0.00	0.05	0.00	0.00	0.00	0.00	0.00	0.00	0.00	0.04	0.00	0.00	0.00	0.00	1.71	0.00	0.00	0.00	0.00	0.00	51.0	53.8
H13-1	KH2-I	9/23/22	0.00	1.81	0.00	0.00	0.54	0.14	0.00	0.00	0.00	4.21	0.00	1.84	0.00	0.00	0.53	0.02	0.00	0.00	0.22	0.00	41.4	48.3

H13-2	KH2-O	9/23/22	0.00	0.00	0.00	0.00	0.00	0.13	0.00	0.12	0.00	0.00	0.00	0.03	0.00	0.00	5.68	0.05	0.00	0.00	2.08	0.00	110.5	106.7
H14-1	KH3-I	9/23/22	0.00	2.31	0.00	0.00	0.32	0.05	0.00	0.04	0.00	0.10	0.00	0.43	0.00	0.00	0.21	0.00	0.00	0.00	0.00	0.00	62.9	40.2
H14-2	KH3-O	9/23/22	0.00	0.09	0.00	0.00	0.00	0.04	0.00	0.00	0.00	0.00	0.00	0.00	0.00	0.00	7.87	0.08	0.00	0.00	1.33	0.00	105.6	90.3
H15-1	OH1-I	9/24/22	0.00	0.00	0.00	0.00	0.20	0.02	0.00	0.00	0.00	0.00	0.00	0.00	0.00	0.00	0.28	0.00	0.00	0.00	0.00	0.00	99.6	91.1
H15-2	OH1-O	9/24/22	0.09	0.17	0.00	0.00	6.05	0.32	0.44	0.32	0.00	0.68	0.00	0.00	0.00	0.00	0.16	0.02	0.00	0.00	0.00	0.00	102.5	103.6
H16-1	OH2-I	9/23/22	0.00	0.14	0.00	0.00	0.44	0.04	0.00	0.00	0.00	0.32	0.00	0.00	0.00	0.00	0.08	0.00	0.00	0.00	0.00	0.00	95.4	79.5
H16-2	OH2-O	9/23/22	0.00	0.00	0.00	0.00	0.00	0.15	0.00	0.15	0.00	0.17	0.00	0.09	0.00	0.00	4.78	0.00	0.00	0.00	0.22	0.00	100.5	87.1
H17-1	OH3-I	9/26/22	0.00	0.08	0.00	0.00	0.00	0.00	0.00	0.00	0.00	0.00	0.00	0.00	0.00	0.00	0.04	0.00	0.00	0.00	0.00	0.00	40.5	64.2
H17-2	OH3-2	9/26/22	0.00	0.03	0.00	0.00	0.12	0.01	0.00	0.00	0.00	0.00	0.00	0.00	0.00	0.00	0.43	0.00	0.00	0.00	0.00	0.00	118.0	104.3
Method Detection Limits (ng/g)			0.10	0.11	0.19	0.07	0.09	0.24	0.16	0.24	0.36	0.15	0.07	0.06	0.17	0.22	0.29	0.25	0.13	0.21	0.27	0.24		

Raw data: Indoor and Outdoor air sample data related to figure S2

Code	**Sample _ID**	**Collection _Date**	**6-Chloronicotinic acid**	**6-Chloronicotinic aldehyde**	**6-Chloro-N-methylnicotinamide**	**Acetamiprid**	**Azoxystrobin**	**Clothianidin**	**Dimethoate**	**Dinotefuran**	**Imidacloprid**	**Imidacloprid desnitro**	**Imidacloprid olefin**	**Imidacloprid urea**	**Indoxacarb**	**Metalaxyl**	**Picoxystrobin**	**Pyraclostrobin**	**Sulfoxaflor**	**Thiacloprid**	**Thiamethoxam**	**Thiamethoxam urea**	**Trifloxystrobin**
H1-I	MH1-I	7/7/22	0.000	0.000	0.000	0.000	0.090	0.646	0.000	0.000	0.094	0.000	0.000	0.000	0.000	0.000	0.000	0.000	0.000	0.000	0.145	0.000	0.000
H1-2	MH1-O	7/7/22	0.000	0.000	0.000	0.000	0.199	0.526	0.000	0.000	0.064	0.024	0.000	0.000	0.000	0.025	0.000	0.000	0.000	0.000	0.086	0.000	0.000
H2-1	MH2-I	7/7/22	0.000	0.000	0.000	0.000	0.598	0.746	0.000	0.000	0.050	0.017	0.000	0.000	0.000	0.000	0.013	0.000	0.000	0.000	0.104	0.000	0.000
H2-2	MH2-O	7/7/22	0.000	0.000	0.000	0.000	0.584	0.272	0.000	0.000	0.043	0.012	0.000	0.000	0.000	0.000	0.009	0.008	0.000	0.000	0.042	0.000	0.000
H4-1	MH4-I	9/1/22	0.000	0.000	0.000	0.000	0.000	0.051	0.000	0.000	0.000	0.000	0.000	0.000	0.000	0.000	0.000	0.000	0.000	0.000	0.000	0.000	0.000
H4-2	MH4-O	9/1/22	0.000	0.000	0.000	0.000	0.007	0.039	0.000	0.000	0.000	0.000	0.000	0.000	0.000	0.000	0.000	0.000	0.000	0.000	0.000	0.000	0.000
H5-1	MH5-I	9/7/22	0.000	0.000	0.000	0.000	0.000	0.057	0.000	0.000	0.000	0.000	0.000	0.000	0.000	0.000	0.000	0.000	0.000	0.000	0.000	0.000	0.000
H5-2	MH5-O	9/7/22	0.000	0.000	0.000	0.000	0.013	0.046	0.000	0.000	0.000	0.000	0.000	0.000	0.000	0.000	0.000	0.000	0.000	0.000	0.000	0.000	0.000
H6-1	MH6-I	9/9/22	0.000	0.000	0.000	0.000	0.010	0.039	0.000	0.000	0.000	0.000	0.000	0.000	0.000	0.000	0.000	0.000	0.000	0.000	0.000	0.000	0.000
H6-2	MH6-O	9/9/22	0.000	0.000	0.000	0.000	0.000	0.050	0.000	0.000	0.000	0.000	0.000	0.000	0.000	0.000	0.000	0.000	0.000	0.000	0.000	0.000	0.000
H7-1	MH7-I	9/9/22	0.000	0.000	0.000	0.000	0.020	0.209	0.000	0.000	0.000	0.000	0.000	0.000	0.000	0.000	0.000	0.000	0.000	0.000	0.069	0.000	0.000

H7-2	MH7-O	9/9/22	0.000	0.000	0.000	0.000	0.118	0.037	0.000	0.000	0.000	0.000	0.000	0.000	0.000	0.000	0.000	0.000	0.000	0.000	0.000	0.000	0.000
H8-1	MH8-I	9/9/22	0.000	0.000	0.000	0.000	0.009	0.017	0.000	0.000	0.000	0.000	0.000	0.000	0.000	0.000	0.000	0.000	0.000	0.000	0.000	0.000	0.000
H8-2	MH8-O	9/9/22	0.000	0.000	0.000	0.000	0.057	0.000	0.000	0.000	0.000	0.000	0.000	0.000	0.000	0.000	0.009	0.000	0.000	0.000	0.189	0.000	0.000
H9-1	MH9-I	9/9/22	0.000	0.000	0.000	0.000	0.030	0.025	0.000	0.000	0.000	0.000	0.000	0.000	0.000	0.000	0.000	0.000	0.000	0.000	0.057	0.000	0.000
H9-2	MH9-O	9/9/22	0.000	0.000	0.000	0.000	0.142	0.032	0.000	0.000	0.000	0.000	0.000	0.000	0.000	0.000	0.000	0.005	0.000	0.000	0.021	0.000	0.000
H10-1	MH10-I	9/22/22	0.000	0.000	0.000	0.000	0.022	0.118	0.000	0.000	0.000	0.000	0.000	0.000	0.000	0.000	0.000	0.000	0.000	0.000	0.088	0.000	0.000
H10-2	MH10-O	9/22/22	0.000	0.000	0.000	0.000	0.018	0.065	0.000	0.000	0.000	0.000	0.000	0.000	0.000	0.000	0.000	0.000	0.000	0.000	0.273	0.000	0.000
H11-1	MH11-I	9/22/22	0.000	0.000	0.000	0.000	0.006	0.040	0.000	0.000	0.000	0.000	0.000	0.000	0.000	0.000	0.000	0.000	0.000	0.000	0.100	0.000	0.000
H11-2	MH11-O	9/22/22	0.000	0.000	0.000	0.000	0.005	0.023	0.000	0.000	0.000	0.000	0.000	0.000	0.000	0.003	0.000	0.000	0.000	0.000	0.039	0.000	0.000
H12-1	KH1-I	9/23/22	0.000	0.000	0.000	0.000	0.014	0.015	0.000	0.000	0.000	0.000	0.000	0.000	0.000	0.000	0.000	0.000	0.000	0.000	0.000	0.000	0.000
H12-2	KH1-O	9/23/22	0.000	0.000	0.000	0.000	0.009	0.016	0.000	0.000	0.000	0.000	0.000	0.000	0.000	0.000	0.000	0.000	0.000	0.000	0.000	0.000	0.000
H13-1	KH2-I	9/23/22	0.000	0.000	0.000	0.000	0.018	0.036	0.000	0.000	0.000	0.000	0.000	0.000	0.000	0.000	0.000	0.000	0.000	0.000	0.000	0.000	0.000
H13-2	KH2-O	9/23/22	0.000	0.000	0.000	0.000	0.023	0.022	0.000	0.000	0.000	0.000	0.000	0.000	0.000	0.000	0.000	0.000	0.000	0.000	0.000	0.000	0.000
H14-1	KH3-I	9/23/22	0.000	0.000	0.000	0.000	0.000	0.000	0.000	0.000	0.000	0.000	0.000	0.000	0.000	0.000	0.000	0.000	0.000	0.000	0.000	0.000	0.000
H14-2	KH3-O	9/23/22	0.000	0.000	0.000	0.000	0.004	0.031	0.000	0.000	0.000	0.000	0.000	0.000	0.000	0.000	0.000	0.000	0.000	0.000	0.000	0.000	0.000
H15-1	OH1-I	9/24/22	0.108	0.000	0.000	0.000	0.000	0.000	0.000	0.000	0.041	0.000	0.000	0.000	0.000	0.000	0.000	0.000	0.000	0.000	0.000	0.000	0.000

H15-2	OH1-O	9/24/22	0.000	0.000	0.000	0.000	0.005	0.000	0.000	0.000	0.000	0.000	0.000	0.000	0.000	0.000	0.000	0.000	0.000	0.000	0.000	0.000	0.000
H16-1	OH2-I	9/24/22	0.074	0.000	0.000	0.000	0.005	0.012	0.000	0.000	0.000	0.000	0.000	0.000	0.000	0.000	0.000	0.000	0.000	0.000	0.000	0.000	0.000
H16-2	OH2-O	9/24/22	0.017	0.000	0.000	0.000	0.004	0.012	0.000	0.000	0.000	0.000	0.000	0.000	0.000	0.000	0.000	0.000	0.000	0.000	0.000	0.000	0.000
H17-1	OH3-I	9/26/22	0.000	0.000	0.000	0.000	0.000	0.019	0.000	0.000	0.000	0.000	0.000	0.000	0.000	0.000	0.000	0.000	0.000	0.000	0.000	0.000	0.000
H17-2	OH3-O	9/26/22	0.000	0.000	0.000	0.000	0.000	0.013	0.000	0.000	0.000	0.000	0.000	0.000	0.000	0.000	0.000	0.000	0.000	0.000	0.000	0.000	0.000
Method Detection Limits (ug/L)=>			0.006	0.007	0.009	0.006	0.014	0.001	0.006	0.006	0.006	0.006	0.012	0.008	0.004	0.006	0.010	0.008	0.004	0.006	0.004	0.008	0.006
